# Aggressive and recurrent ovarian cancers upregulate ephrinA5, a non-canonical effector of EphA2 signaling duality

**DOI:** 10.1038/s41598-021-88382-6

**Published:** 2021-04-23

**Authors:** Joonas Jukonen, Lidia Moyano-Galceran, Katrin Höpfner, Elina A. Pietilä, Laura Lehtinen, Kaisa Huhtinen, Erika Gucciardo, Johanna Hynninen, Sakari Hietanen, Seija Grénman, Päivi M. Ojala, Olli Carpén, Kaisa Lehti

**Affiliations:** 1 Translational Cancer Medicine Research Program, University of Helsinki, 00140 Helsinki, Finland; 2 Department of Microbiology, Tumor and Cell Biology, Karolinska Institutet, 171 77 Stockholm, Sweden; 3 Individualized Drug Therapy Research Program, University of Helsinki, 00140 Helsinki, Finland; 4 Institute of Biomedicine, University of Turku, 20520 Turku, Finland; 5 Department of Obstetrics and Gynecology, Turku University Hospital, University of Turku, 20521 Turku, Finland; 6 Department of Biomedical Laboratory Science, Norwegian University of Science and Technology, 7491 Trondheim, Norway

**Keywords:** Ovarian cancer, Cell signalling, Tumour biomarkers

## Abstract

Erythropoietin producing hepatocellular (Eph) receptors and their membrane-bound ligands ephrins are variably expressed in epithelial cancers, with context-dependent implications to both tumor-promoting and -suppressive processes in ways that remain incompletely understood. Using ovarian cancer tissue microarrays and longitudinally collected patient cells, we show here that ephrinA5/*EFNA5* is specifically overexpressed in the most aggressive high-grade serous carcinoma (HGSC) subtype, and increased in the HGSC cells upon disease progression. Among all the eight ephrin genes, high *EFNA5* expression was most strongly associated with poor overall survival in HGSC patients from multiple independent datasets. In contrast, high *EFNA3* predicted improved overall and progression-free survival in The Cancer Genome Atlas HGSC dataset, as expected for a canonical inducer of tumor-suppressive Eph receptor tyrosine kinase signaling. While depletion of either *EFNA5* or the more extensively studied, canonically acting *EFNA1* in HGSC cells increased the oncogenic EphA2-S897 phosphorylation, *EFNA5* depletion left unaltered, or even increased the ligand-dependent EphA2-Y588 phosphorylation. Moreover, treatment with recombinant ephrinA5 led to limited EphA2 tyrosine phosphorylation, internalization and degradation compared to ephrinA1. Altogether, our results suggest a unique function for ephrinA5 in Eph-ephrin signaling and highlight the clinical potential of ephrinA5 as a cell surface biomarker in the most aggressive HGSCs.

## Introduction

Among epithelial ovarian cancers (OC), high-grade serous carcinoma (HGSC) is the most common and aggressive subtype with 5-year survival remaining as low as 36–48%^1–3^. Frequently, HGSC cases are diagnosed at an advanced stage characterized by cancer cell dissemination into the peritoneal fluid coupled with ascites formation and solid metastases in peritoneal/abdominal organs^3,4^. The main treatment regimen for HGSC is debulking surgery combined with platinum-based chemotherapy, which can be further supported by targeted treatments, such as poly ADP-ribose polymerase (PARP) inhibitors and bevacizumab (vascular endothelial growth factor blocking antibody)^5–7^. The addition of these targeted therapies in the treatment repertoire has increased survival in certain subgroups of HGSC patients, but the large majority still recur, progressively develop treatment resistance, and succumb to the disease^8–10^. Therefore, understanding HGSC aggressiveness is essential for further progress towards the development of new improved biomarkers and therapeutic strategies.

Erythropoietin producing hepatocellular (Eph) receptors, the largest family of receptor tyrosine kinases (RTKs), and their ligands ephrins are involved in a variety of conditions in both physiology and disease^11^. The canonical Eph receptor signaling occurs via interactions with the ephrin ligands. In cancer, Eph receptors are, however, frequently upregulated in conjunction with ephrin downregulation, thus impairing receptor-ligand binding^12^. Eph receptors are divided into two subclasses depending on the preference for their ligands: Generally, EphAs bind glycosylphosphatidylinositol (GPI) membrane-anchored A-class ephrin ligands, and EphBs bind transmembrane B-class ephrin ligands^13,14^. The signaling mediated by the ephrins and Ephs is context-dependent, and distinct modes of Eph receptor activation (ligand-dependent and -independent) and signaling direction (forward, in the Eph-expressing cell, and reverse, in the ephrin-expressing cell) promote different downstream signaling outcomes (tumor-suppressive vs pro-tumorigenic)^11,15^. In distinct OC datasets, this context-dependent signaling has been linked to different clinical outcomes based on, for instance, association of high *EPHA2* and *EPHA4* expression with poor patient survival^16–26^. Among their ligands, opposing clinical associations have instead been reported for *EFNA1* and *EFNA5*^16,18^.

The widely studied Eph in cancer, EphA2, has been proposed as therapeutic target in OC, albeit none of the reported strategies have provided promising results in clinical trials^27^. Upon interaction with ephrinA ligand, EphA2 transphosphorylation at specific cytoplasmic tyrosine residues leads to the receptor activation, followed by internalization of the receptor-ligand complex and eventually receptor degradation or dephosphorylation^12,28^. This signaling mode has been mostly related to the anti-invasive and growth-suppressive cancer cell functions. Ligand independently, EphA2 can mediate tumor-promoting signaling via crosstalk with various growth factor receptors, which through AGC family kinases (Akt, PKA and RSK) promote the phosphorylation of EphA2 S897 residue^29–31^. The pro-invasive EphA2 activity can also be regulated upon Src-kinase activation and limited cleavage by matrix metalloproteinase MT1-MMP^32^. Moreover, we have found that, rather than the depletion of total EphA2 activity, inhibition of the EphA2-pS897 signaling and coincident restoration of the canonical EphA2-pY588 are coupled with effective HGSC cell sensitization to platinum chemotherapy^29^. Therefore, in addition to targeting Eph receptors, altering the signaling elicited by the ephrin ligands can help to revert the tumor-promoting signaling to a suppressive state^33,34^.

High *EFNA5* expression has been linked to poor survival in OC patients^18,20,21^ although high ephrin levels in general are more frequently associated to favorable clinical outcome due to the transduction of tumor-suppressive signals upon EphA2-Y588 phosphorylation^12,31^. Intrigued by our observation of the opposite clinical association of *EFNA5* and *EFNA3* in OC TCGA data, we sought to investigate *EFNA5* and the corresponding protein ephrinA5 clinically and functionally in HGSC. We find here that, in addition to the strong association of high *EFNA5* mRNA with poor HGSC patient survival, ephrinA5 protein is specifically upregulated in the most aggressive HGSC tumors compared to the other histopathological OC subtypes. Functionally, our results describe a unique signaling pattern for ephrinA5 that, divergent from the more extensively studied ephrinA1, can even reduce the canonical EphA2 phosphorylation at Y588. Altogether, our findings highlight the clinical potential of ephrinA5 as a biomarker and a possible treatment target in HGSC.

## Results

### High *EFNA5* expression correlates with poor HGSC patient survival

**Figure 1:**
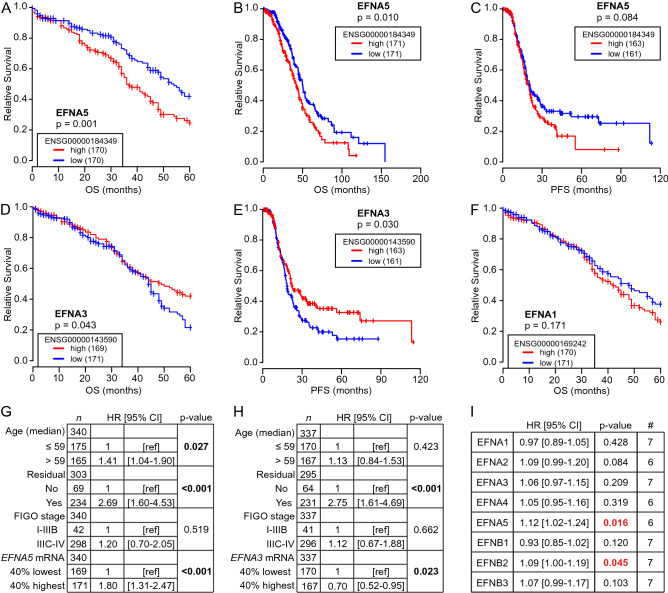
Ephrin and Eph gene expression are diversely associated to survival in HGSC. (**A**–**F**) Kaplan–Meier survival curves illustrate the 5-year and 13-year overall survival (OS) or the progression-free survival (PFS) of patients with high or low (top 40% vs bottom 40%) *EFNA5* (**A**: 5-y OS, **B**: 13-y OS, **C**: PFS), *EFNA3* (**D**: 5-y OS, **E**: PFS), and *EFNA1* (**F**: 5-y OS) in TCGA HGSC dataset. Logrank test was used. (**G**, **H**) Multivariate analysis results for *EFNA5* (**G**) and *EFNA3* (**H**) OS associations when considering the variables age at diagnosis (median cutoff of 59 years), residual tumor after surgery (no vs yes) and FIGO stage (I–IIIB vs IIIC–IV). Cox regression was used. HR [95% CI] = hazard ratio with 95% confidence interval. (**I**) Overall hazard ratios and *p* values from the Cox regression tests performed to analyze ephrin survival associations in HGSC using the *curatedOvarianData* database. Significant associations between poor OS and high ligand expression are indicated in red. # = number of included datasets.

Unbiased analyses of different OC patient cohorts have revealed survival associations for several genes encoding Eph receptors (*EPHA/B*) and ephrin ligands (*EFNA/B*), including contradictory results^16,18,20–22,24^. To systematically investigate all the 14 Eph receptors and 8 ephrin ligands in clinical HGSC, we analyzed overall survival (OS) and progression-free survival (PFS) in patients with 40% highest versus 40% lowest gene expression for all the Ephs and ephrins first using TCGA HGSC mRNA dataset^35^ (see Supplementary Fig. 1A for cohort clinical data summary). High *EFNA5* was associated to poor 5-year OS (Fig. 1A; Supplementary Fig. 1B; *p* = 0.001). This association remained significant with extended survival time analysis (13-year; Fig. 1B; Supplementary Fig. 1C; *p* = 0.010) as well as when *k*-means clustering was used for patient grouping (Supplementary Fig. 1B; *p* = 0.003), and high *EFNA5* also showed a trend to shorter time to recurrence/PFS (Fig. 1C). In contrast, high *EFNA3* correlated with favorable 5-year OS (Fig. 1D; *p* = 0.043) as well as longer PFS (Fig. 1E; *p* = 0.030), whereas *EFNA1* and the other ephrin ligands did not correlate with patient survival (Fig. 1F; Supplementary Fig. 1B, C). The associations of *EFNA5* (*p* < 0.001; hazard ratio (HR) = 1.80, indicating association to poor OS) and *EFNA3* (*p* = 0.023; HR = 0.70, indicating association to favorable OS) remained significant when integrating age at diagnosis, residual tumor after surgery and FIGO stage in a Cox multivariate analysis (Fig. 1G, H).

Out of the 14 Eph receptors, only high *EPHA2* and *EPHA4* correlated significantly with poor 5-year OS (Supplementary Fig. 1D, E; *p* < 0.05), whereas high *EPHA1* correlated with favorable OS (Supplementary Fig. 1B, C and F; *p* ≤ 0.040). *EPHA2* association to poor OS remained significant in Cox multivariate analysis (Supplementary Fig. 1G; *p* = 0.044, HR = 1.36).

For result validation, we next used seven independent OC datasets (n = 815 HGSC patients) from a publicly available database with pooled mRNA data from various OC studies (*curatedOvarianData*^36^). Across these datasets as well as in TCGA, the genes for ephrin ligands were variably expressed; *EFNA1*, *EFNB1* and *EFNB2* showing relatively high and *EFNA2* low global expression (Supplementary Fig. 2A, B). The overall expression levels of the receptors *EPHA1*, *EPHA2*, *EPHA4* and *EPHB2* were higher than those of *EPHA3*, *EPHA5*, *EPHA6*, *EPHA7*, *EPHA8*, *EPHA10* and *EPHB1* (Supplementary Fig. 2A, B).

**Figure 2:**
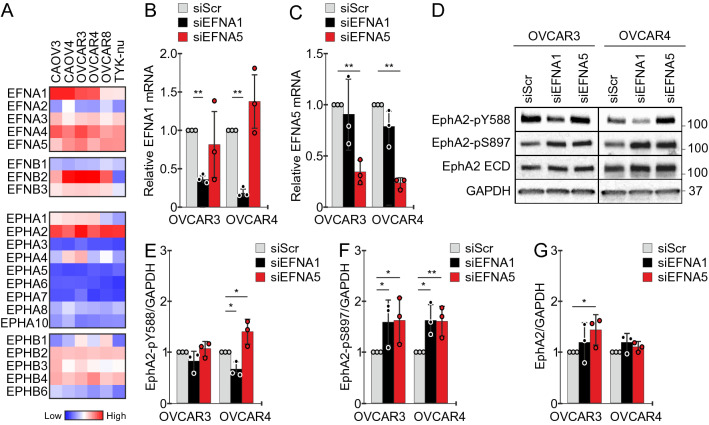
Canonical EphA2-pY588 activation remains unaltered or is even increased after depletion of *EFNA5*. (**A**) Heat map for ephrin ligands and Eph receptor mRNAs in selected HGSC cell lines. Data obtained from CCLE and illustrated per cell line. (**B**, **C**) Quantification of *EFNA1* (**B**) and *EFNA5* (**C**) mRNAs in OVCAR3 and OVCAR4 with silenced *EFNA1* or *EFNA5* compared to the controls. N = 3. siScr is set to one. (**D**–**G**) EphA2 (total and phosphorylated) in OVCAR3 and OVCAR4 after silencing of *EFNA1* or *EFNA5* (**D**) along with EphA2-pY588 (**E**), EphA2-pS897 (**F**), and EphA2 (**G**) quantifications. N = 3. siScr is set to one. Full-length blots are presented in Supplementary Fig. 5. *p* values (Student’s t-test): *< 0.05; **< 0.01.

With these OC datasets, we performed multivariate analyses for OS, considering also residual tumor after surgery and FIGO stage. The ligand encoding genes *EFNA5* and *EFNB2* correlated with poor OS (HR > 1.00, *p* < 0.05) in the combined validation datasets (Fig. 1I, Supplementary Fig. 3A, B). The receptor genes *EPHA2*, *EPHB2* and *EPHB4* also correlated with poor OS, whereas *EPHA1* correlated with favorable OS (Supplementary Fig. 4A–E). Among all EFNs and EPHs, *EFNA5* was one of the three genes with strongest predictive power for poor OS, showing the strongest association of all the ligands to OS in the multivariate analysis (Supplementary Figs. 3, 4).

### Unlike *EFNA1,* endogenous* EFNA5* is not a canonical EphA2-pY588 signaling mediator in ovarian cancer cells

EphrinA-mediated EphA2 receptor tyrosine kinase activation is linked to tumor-suppressive signaling, and thus disfavored in cancer cells^12,37,38^. Considering the inverse contribution of *EFNA5* (encoding ephrinA5) to cancer aggressiveness and poor clinical HGSC outcome, we sought to further examine ephrinA5 in HGSC. First, to identify a suitable functional cell model, we assessed mRNA levels for ephrins and Eph receptors in six human *TP53* mutant HGSC cell lines^39^. All ephrin genes except *EFNA2* and *EFNB1* were expressed variably, *EFNA5* being relatively high in all these cell lines (Fig. 2A; the Cancer Cell Line Encyclopedia, CCLE, https://portals.broadinstitute.org/ccle). All the six HGSC cells were characterized by particularly high *EPHA2*, whereas the other Ephs were variably expressed and/or low (Fig. 2A) in a relatively similar manner as in the patients (see Supplementary Fig. 2).

To compare the effects of ephrinA1 and ephrinA5 in EphA2 activation/phosphorylation, we used the epithelial HGSC cell lines OVCAR3 and OVCAR4^29^, which both expressed these three genes, for *EFNA1* and *EFNA5* gene knockdown. Using specific pooled siRNAs, the ephrins were silenced with > 70% efficiency (Fig. 2B, C). In both cell lines, EphA2 was constitutively phosphorylated at Y588 and S897 amino acid residues (Fig. 2D). Notably, while *EFNA1* depletion clearly reduced tumor-suppressive EphA2-pY588, as expected upon depletion of the ligand-mediated signaling, *EFNA5* silencing left EphA2-pY588 essentially unaltered in OVCAR3 and even increased this tyrosine phosphorylated receptor in OVCAR4 (Fig. 2D, E). Compared to control cells, the oncogenic EphA2-pS897 was increased after silencing of either *EFNA1* or *EFNA5* (Fig. 2D, F), whereas total EphA2 was less affected, or increased after *EFNA5* silencing in OVCAR3 (Fig. 2D, G). These results suggest that while endogenous ephrinA1 mediates the canonical EphA2-Y588 phosphorylation, ephrinA5 has a different function with potential to instead limit this tumor-suppressive EphA2-pY588.

### EphrinA5 is an inefficient activator of EphA2-pY588 signaling and receptor internalization compared to ephrinA1

**Figure 3:**
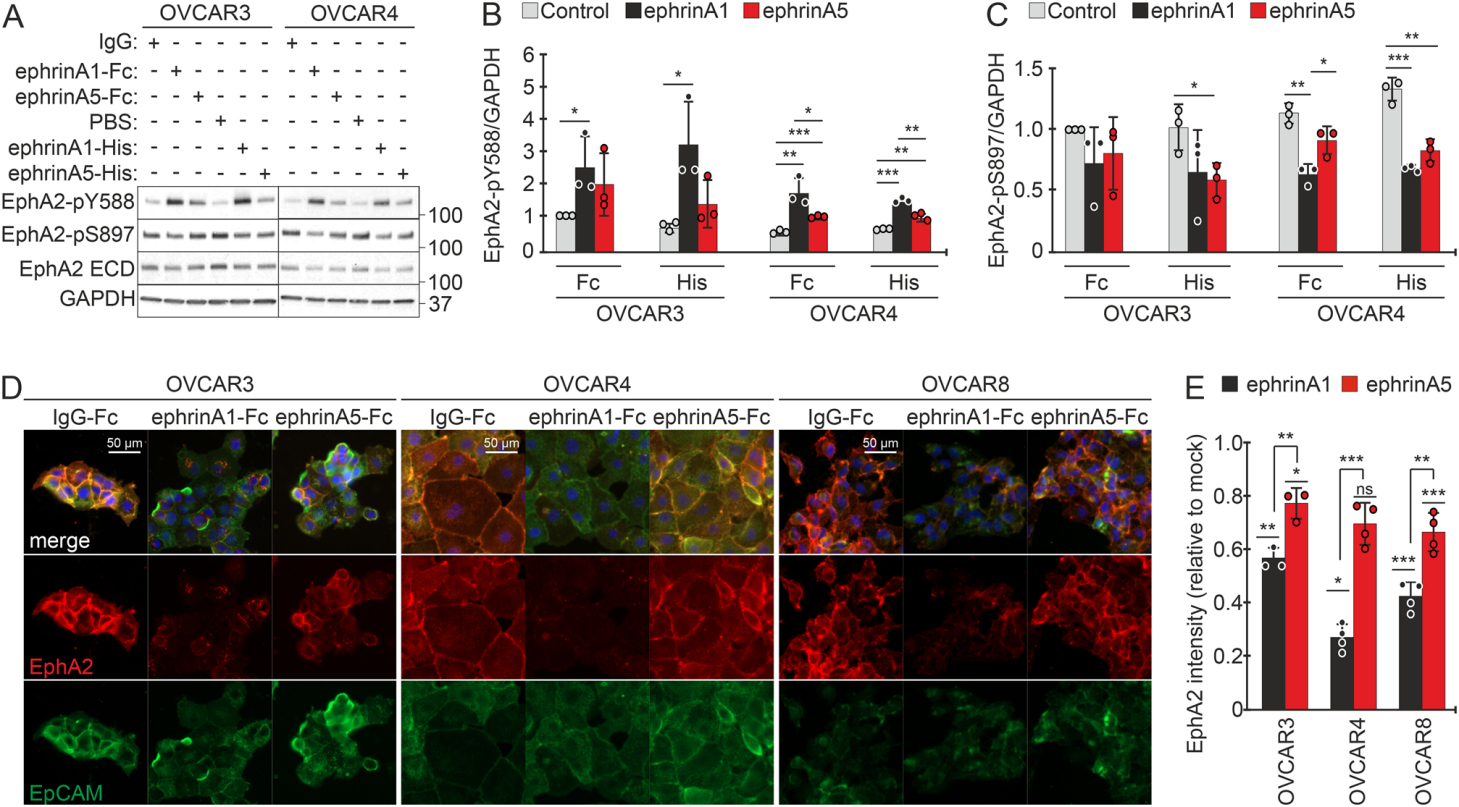
EphA2 activation by ephrinA5 results in weaker canonical EphA2-pY588 signaling than upon ephrinA1-mediated activation. (**A**–**C**) EphA2 (total and phosphorylated at Y588 or S897) in OVCAR3 and OVCAR4 after treatment with soluble recombinant dimeric (Fc-tagged) and monomeric (His-tagged) ephrinA1 and ephrinA5 for 120 min were assessed by immunoblotting (**A**) and quantified for EphA2-pY588 (**B**) and EphA2-pS897 (**C**). N = 3. Full-length blots are presented in Supplementary Fig. 5. (**D**, **E**) Fluorescent micrographs of EphA2 (red) and EpCAM (green) in ephrinA1-Fc and ephrinA5-Fc treated OVCAR3, OVCAR4, and OVCAR8 cells (**D**) and corresponding EphA2 quantification (**E**). N = 3 (OVCAR3) or 4 (OVCAR4 and OVCAR8). Mock is set to one. *p* values (Student’s t-test): ns = not significant, *< 0.05; **< 0.01; ***< 0.001.

To better understand the unexpected contribution of ephrinA5 to EphA2 signaling, OVCAR3 and OVCAR4 were treated with dimeric (Fc-tagged) and monomeric (His-tagged) recombinant ephrinA1 and ephrinA5 for 120 min. Both ephrinA1 and ephrinA5 were detected bound to the cells after the treatment (Supplementary Fig. 5B). As described above, EphA2 was constitutively phosphorylated both at Y588 and S897 residues in OVCAR3 and OVCAR4 (Fig. 3A). While treatment with ephrinA1 (Fc- and His-tagged) increased the tumor-suppressive EphA2-pY588 in these cells, as expected upon ligand-mediated receptor activation, this phosphorylation was not altered by ephrinA5 (Fc- and His-tagged) in OVCAR3 and remained lower in OVCAR4, compared to the respective ephrinA1 (Fc- and His-tagged) treated cells (Fig. 3A, B). The oncogenic EphA2-pS897 was reduced by ephrinA1 (Fc- and His-tagged) in OVCAR4, and by ephrinA5-His treatment in both cells (Fig. 3A, C). Total EphA2 remained unaltered/unaffected upon treatments with the exogenous ligands under these experimental conditions, except for a reduction upon ephrinA1-His treatment in OVCAR4 (Fig. 3A; Supplementary Fig. 5C).

To elucidate putative differences in EphA2 internalization occurring after activation by ephrinA1 or ephrinA5, we assessed the localization of this receptor by immunofluorescence in HGSC cells after treatment with the dimeric Fc-tagged ephrins for 45 min (treatment time adjusted to efficient EphA2 activation by the dimeric ephrins^40^). Prominent cell surface localization of EphA2 detected in control cells was lost and the total EphA2 intensity reduced in OVCAR3, OVCAR4 as well as in the third, more mesenchymal HGSC cell line, OVCAR8^29^, after ephrinA1 treatment (Fig. 3D, E), indicating EphA2 internalization and degradation. Markedly, ephrinA5 treatment had minor effects on EphA2 localization and resulted in only slightly reduced signal intensity in OVCAR3 and OVCAR8 (Fig. 3D, E). Of note, EphA2 intensity remained significantly higher in these HGSC cells after ephrinA5 treatment compared to ephrinA1 treatment (Fig. 3D, E; OVCAR3: *p* = 0.009, OVCAR4: *p* < 0.001, OVCAR8: *p* = 0.002).

### EphrinA5 is expressed in the cancer cells and strongly associates with the most aggressive HGSC subtype of ovarian cancer

**Figure 4:**
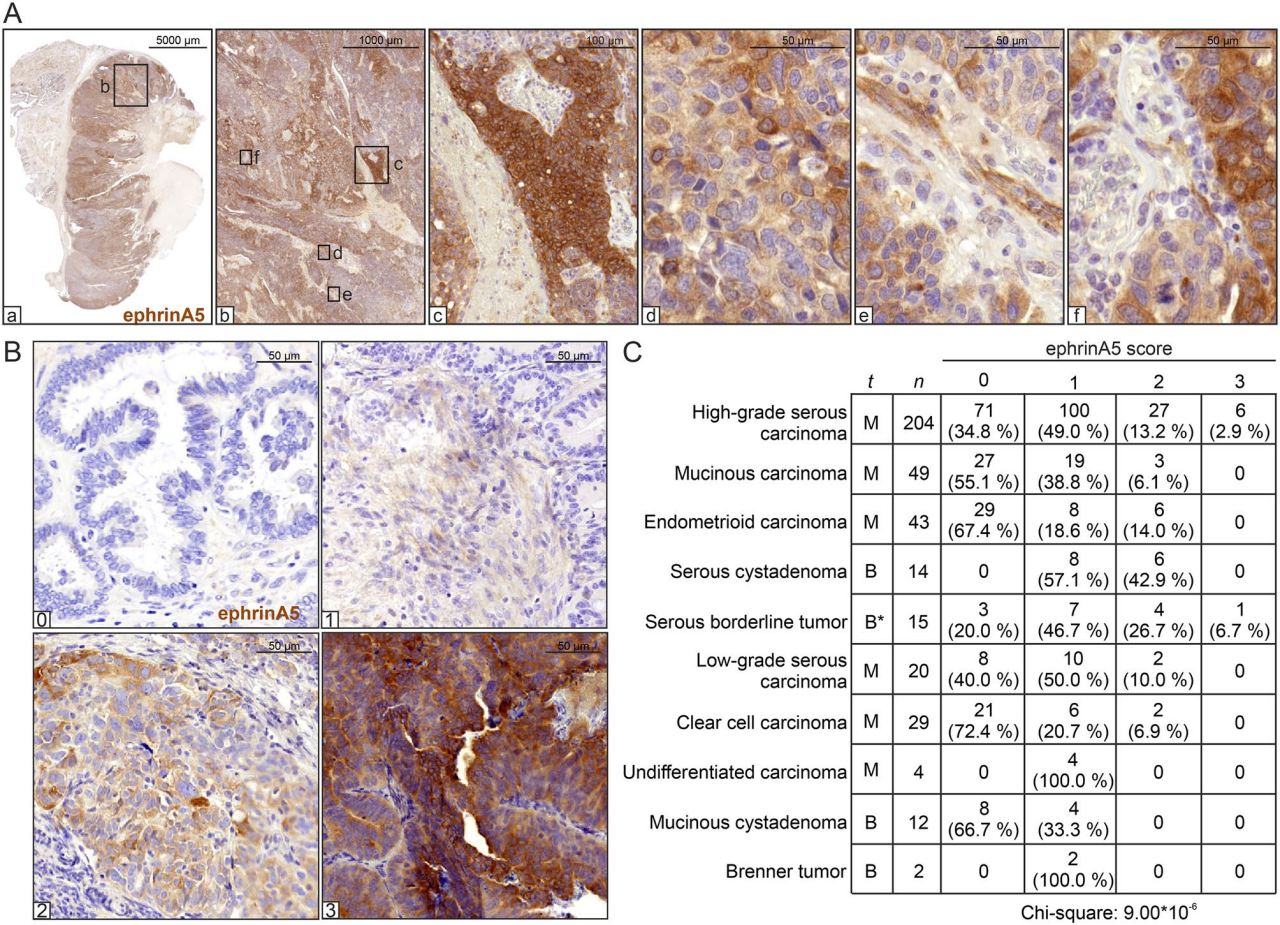
EphrinA5 protein expression is heterogeneous and associates with disease malignancy. (**A**) EphrinA5 IHC of a diagnosis-stage, treatment-naïve, primary HGSC tumor. Magnified images for different tumor areas. (**B**) Representative images of ephrinA5 IHC used for scoring the ovarian tumor TMAs. (**C**) Correlation of ephrinA5 protein expression with different epithelial OC subtypes and benign tumors ordered according to the weighted arithmetic mean of ephrinA5 score (descending order). t = tissue type, M = malignant, B = benign, * = mostly benign. Pearson chi‐square test showed highest overall ephrinA5 expression in HGSC (p *=* 9.00 × 10^−6^).

To assess the expression of the non-canonically acting ephrinA5 ligand in clinical OC tumors, we first analyzed this protein in different tissue compartments of a large treatment-naïve, primary HGSC section by immunohistochemistry (IHC). Compared to the surrounding tissue, ephrinA5 levels were generally stronger in the HGSC cells, showing patterns of segregated, high and relatively lower ephrinA5 expressing tumor areas (Fig. 4A, a–d). The tumor stroma showed mainly negative to low expression with some ephrinA5 positivity in vessel-like structures (Fig. 4A, e–f).

To investigate the relevance of this ephrin ligand among the different OC subtypes, we assessed ephrinA5 expression in 392 epithelial OC and benign tissue samples by IHC of tissue microarrays (TMAs). See examples for grading from negative to strong expression in Fig. 4B. Among all epithelial OC subtypes and benign tissues, HGSC comprising the most aggressive tumors had the highest overall ephrinA5 expression (Fig. 4C; *p* = 9.00 × 10^−6^), supporting the strong association between ephrinA5 and OC malignancy.

### EphrinA5 increases during disease progression in patient-derived HGSC cells

**Figure 5:**
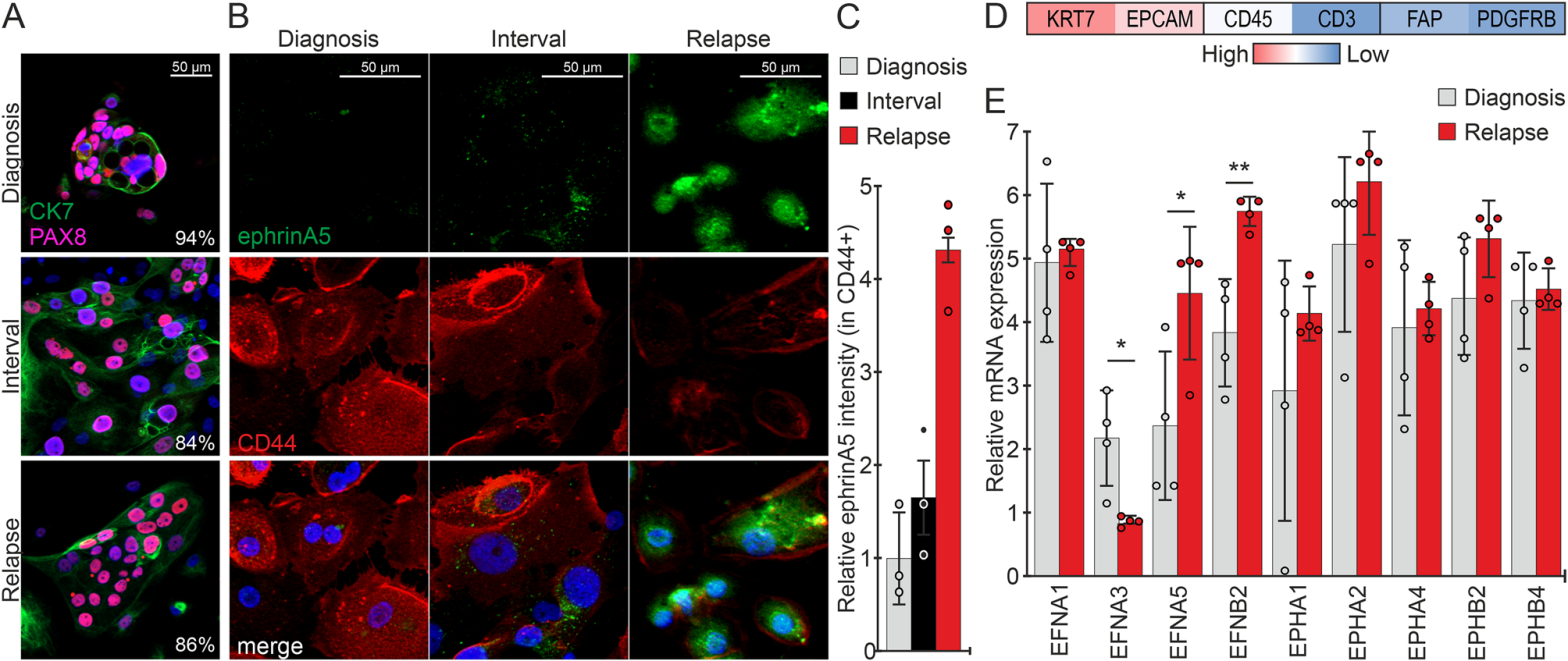
EphrinA5 increases upon HGSC progression in patient-derived HGSC cells. (**A**) Fluorescent micrographs of cytokeratin-7 (CK7, green) and PAX8 (pink) in cancer cells isolated from HGSC patient ascites at the time of diagnosis, interval and relapse. Percentage of PAX8 positivity (average of 3 images/clinical time point) is indicated in the micrographs. N = 1 patient, 3 clinical time points. (**B**, **C**) Fluorescent micrographs of ephrinA5 (green) and CD44 (red) in the set of longitudinal HGSC patient-derived ascites cells shown in A (**B**) and corresponding ephrinA5 quantification in CD44+ cells (**C**). N = 1 patient, 3 clinical time points (**B**), 3 images/clinical time point with 91–215 ascites-derived cells per image (**C**). Intensity at diagnosis is set to one. (**D**) Heatmap of averaged mRNA expression for the epithelial (*KRT7*, *EPCAM*), immune cell (*CD45*, *CD3*) and fibroblast (*FAP*, *PDGFRB*) markers in samples included in (**E**). (**E**) Chart illustrates changes in *EFNA1*, *EFNA3*, *EFNA5*, *EFNB2*, *EPHA1*, *EPHA2*, *EPHA4*, *EPHB2* and *EPHB4* mRNAs in HGSC patient-derived ascites cells from the time of diagnosis to relapse stage. N = 4 samples/clinical time point. *p* values (Student’s t-test): *< 0.05; **< 0.01.

To investigate the changes in ephrinA5 expression upon disease progression, considering the prominent protein expression particularly in the cancer cells, we collected ascites cells longitudinally from a HGSC patient at three time points including diagnosis, interval (after three rounds of platinum chemotherapy) and relapse. The isolated cells were 84–94% positive for the nuclear HGSC marker PAX8 (Fig. 5A). Immunofluorescence of ephrinA5 coupled with the cell surface marker CD44 allowed us to investigate the expression of this ligand in the samples enriched for cancer cells. EphrinA5 was essentially undetectable in this diagnosis sample and expressed at low levels in HGSC cells from the chemotherapy-treated interval time point of the same patient (Fig. 5B). Notably, cells from the relapse time point had higher expression of ephrinA5 compared to cells from the earlier diagnosis and interval stages of this patient’s longitudinal samples (Fig. 5B, C).

To uncover more comprehensive expression changes of the ephrins and Eph receptors upon HGSC progression, we analyzed RNA sequencing data of ascites samples from four HGSC patients at the times of diagnosis and relapse of the disease. These samples were characterized by high expression of the epithelial markers *KRT7* and *EPCAM*, and lower to very low expression of immune cell (*CD45, CD3*) and fibroblast (*FAP*, *PDGFRB)* markers (Fig. 5D). In contrast to decreased *EFNA3* expression, *EFNA5* was significantly increased from the time of diagnosis to relapse (Fig. 5E; *EFNA3*: *p* = 0.015, *EFNA5*: *p* = 0.038). In these same HGSC samples, *EFNB2* was likewise increased, whereas *EFNA1*, *EPHA1*, *EPHA2*, *EPHA4*, *EPHB2* and *EPHB4* expression remained essentially unaltered (Fig. 5E; *EFNB2*: *p* = 0.005). These results correlate with the above survival associations, where low *EFNA3* showed association to short PFS and high *EFNA5* and *EFNB2* were associated with poor OS (see Fig. 1). Altogether, these data suggest that the uniquely acting ephrinA5 can contribute to the abysmal HGSC patient outcome upon upregulation in the malignant HGSC cells and during progression of the disease.

## Discussion

The tumor-suppressive signaling elicited by EphA2-ephrinA complexes is often halted in aggressive cancers via EphA2 receptor overexpression coupled with ephrinA ligand downregulation^12^. Yet, the epithelial OC cells display variable ephrinA/*EFNA* expression (see Fig. 2A) and high mRNA expression of *EFNA5,* encoding the ephrinA5 ligand, has been repeatedly associated to poor survival in OC patients^18,20,21^. Here we show that ephrinA5 protein levels are high specifically in the most aggressive HGSC and further upregulated transcriptionally upon disease progression. Functionally, we describe an unusual signaling pattern for this ligand in HGSC cells, whereby it leaves unaffected or even impairs the canonical, tumor-suppressive tyrosine phosphorylation and subsequently induced internalization and degradation of EphA2 receptor.

Unbiased analyses of independent OC cohorts have revealed survival associations of individual Eph and ephrin genes, resulting in an incomplete understanding of the clinical importance of the distinct receptors and ligands in this disease^16,18,20–22,24^. Here we report that, in line with the canonical function of ephrins in promoting tumor-suppressive Eph receptor signaling^12^, low *EFNA3* mRNA levels are associated to poor OS and short PFS in the TCGA HGSC dataset. In contrast, high *EFNA5* expression is associated to poor survival in HGSC. In agreement with previous reports^16–18,20–22^, our systematic analysis of all the 22 genes for Eph receptors (*EPH*s) and ephrin ligands (*EFN*s) concurs with the association of high *EFNB2*, *EPHA2, EPHB2* and *EPHB4* with poor OS. For the Ephs/ephrins with previously reported inconsistent findings^16,18,22,24^, our analysis supports the correlation of low *EPHA1* mRNA expression with poor OS, whereas the analyzed data lacked significant association for *EFNA1*.

Recurrence and development of treatment resistance in HGSC patients remains an unmet clinical need and thus understanding these processes at the functional level is essential to provide new therapeutic opportunities. We show here that ephrinA5 protein as well as mRNA expression in ascites-derived HGSC cells was increased in post-treatment samples upon disease progression. Previous studies have explored the interactions of EphA2 with ephrinA1 and ephrinA5 and the corresponding crystal structures for these Eph/ephrin complexes have been resolved^41–43^. However, the signaling mechanisms elicited by endogenous ephrinA5 in cancer cells as well as the differences between ephrinA1- and ephrinA5-mediated EphA2 receptor activation have remained elusive. Further studies will be of interest to fully understand the complex Eph/ephrin signaling mechanisms and outputs, with possible heterotypic Eph-Eph and Eph-growth factor receptor crosstalk, alternative binding of ephrinA ligands to EphB receptors (including ephrinA5-EphB2 interaction, as occurs in neural development) and vice versa (e.g. ephrinB2-EphA4, as reported in the context of monocyte adhesion to endothelial cells), proteolytic ligand and/or receptor cleavages, as well as ephrin ligand crosstalk with other RTKs^28,44,45^. Nonetheless, our current results provide evidence indicating that ephrinA5 can hinder the tumor suppressive signaling normally coupled with degradation of EphA2, thus allowing the oncogenic receptor to function at the HGSC cell membranes. We have recently described a strategy to sensitize HGSC cells to chemotherapy by blocking the platinum-induced, pro-tumorigenic EphA2-pS897 signaling and simultaneously restoring EphA2 phosphorylation at Y588^29^. Disrupting the balance of the canonically acting ephrinAs and the tumor promoting ephrinA5 could likewise help to restore the tumor-suppressive signaling through EphA2-Y588 phosphorylation in order to reduce tumor malignancy and development of the increasingly therapy resistant relapses.

In this study we describe a specific link between high ephrinA5 protein expression and HGSC, the most aggressive epithelial OC subtype, and show that ephrinA5 increases upon disease progression and associates to poor survival. We further report a non-canonical signaling function for ephrinA5 in HGSC cells, whereby, opposite to the tumor-suppressive signaling elicited by EphA2-ephrinA1 complexes, ephrinA5 even limits the canonical activation of EphA2 through phosphorylation at Y588. Our findings suggest the potential use of ephrinA5 as an indicator of disease subtype and progression stage, as well as its relevance as a putative survival biomarker in HGSC.

## Methods

### Antibodies and reagents

The following antibodies were used: anti-CD44/HCAM (sc-7297, Santa Cruz, immunofluorescence (IF): 1:400), anti-CK7 (YM3054, ImmunoWay, IF: 1:600), anti-EpCAM (#2929, Cell Signaling Technologies, IF: 1:800), anti-EphA2 ECD (AF3035, R&D Systems, immunoblot (IB): 1:1000, IF: 1:100), anti-EphA2-pS897 (#6347, Cell Signaling Technologies, IB: 1:1000), anti-EphA2-pY588 (#12677, Cell Signaling Technologies, IB: 1:1000), anti-ephrinA1 (sc-911, Santa Cruz, IB: 1:1000), anti-ephrinA5 (ab60705, Abcam, IB: 1:1000, IF: 1:100, immunohistochemistry (IHC): 1:200), anti-ephrinA5 (38-0400, Life Technologies, IF: 1:100), anti-GAPDH (G8795, Sigma-Aldrich, IB: 1:10000), anti-PAX8 (10336-1-AP, Proteintech, IF: 1:100), anti-mouse HRP-conjugate (P0260, Dako, IB: 1:2000), anti-rabbit HRP-conjugate (P0448, Dako, IB: 1:2000), anti-goat HRP-conjugate (P0449, Dako, IB: 1:2000), anti-goat Alexa Fluor 568 conjugate (A11057, Thermo Scientific, IF: 1:500), anti-mouse Alexa Fluor 488-conjugate (A21202, Life Technologies, IF: 1:500), anti-rabbit Alexa Fluor 568-conjugate (A11036, Thermo Scientific, IF: 1:500). For mounting, Vectashield with 4′,6-diamidine-2-phenylindole dihydrochloride (DAPI; H-1200, Vector Laboratories) was used. The following recombinant proteins were used: ephrinA1-Fc (6417-A1, R&D Systems, 1 µg/ml), ephrinA1-His (10,882-H08H, Life Technologies, 1 µg/ml), ephrinA5-Fc (374-EA, R&D Systems, 1 µg/ml), ephrinA5-His (10,192-H08H, Life Technologies, 1 µg/ml), and IgG-Fc (110-HG, R&D Systems, 1 µg/ml).

### Cell lines

Human HGSC cell lines OVCAR3, OVCAR4, and OVCAR8 (National Cancer Institute, U.S.) were cultured in RPMI-1640 (Sigma-Aldrich) supplemented with 10% fetal bovine serum (Gibco), 1% penicillin and streptomycin (Gibco), 1% glutaMAX (Gibco), and 10 µg/ml human insulin (OVCAR3 only, Sigma-Aldrich). Cells were cultured according to manufacturer´s instructions and checked routinely for mycoplasma contamination using MycoAlertPlus Mycoplasma Detection Kit (Lonza).

### Patient samples

All studies involving clinical material were performed in accordance with the ethical standards laid in the 1975 Declaration of Helsinki. Each patient gave written informed consent, and tissue and ascites specimens were collected from consented patients at the Department of Obstetrics and Gynecology, Turku University Central Hospital. The study protocol and use of all material was approved by The Ethics Committee of the Hospital District of Southwest Finland (ETMK: ETMK145/1801/2015) and the Hospital District of Helsinki and Uusimaa (HUS: HUS359/2017).The clinical material was collected under the auspices of Auria Biobank.

Treatment-naïve, primary HGSC tumor was collected at the time of debulking surgery and subsequently formalin-fixed and paraffin-embedded to later be used for immunohistochemical stainings.

Fresh patient-derived ascites fluid was collected at the time of diagnosis and longitudinally at interval and relapse-stages and processed for ex vivo cultures as described by Moyano-Galceran et al^29^. Longitudinal ascites-derived cells were simultaneously grown on glass coverslips and stained by immunofluorescence to assess cancer cell purity.

### RNA sequencing

Ascites-derived cells from diagnosis and relapse-stage (n = 4 samples/clinical time point) were used for paired-end RNA-sequencing. Total RNA was extracted from fresh ascites-derived cells by using RNeasy kit (Qiagen) with DNase I treatment according to manufacturer’s instructions. RNA quality and concentration were tested by Bioanalyzer 2100 (Agilent). Paired-end 100 bp RNA-seq producing around 60M reads was carried out on Illumina HiSeq4000 platform and the data was processed using SePIA, a comprehensive RNA-seq data processing workflow^46^. Read pairs were trimmed using Trimmomatic, and trimmed reads were aligned to the reference genome (GRCh38.d1.vd1) using STAR (version 2.5.2b), allowing up to 10 mismatches, and all alignments for a read were output^47^. Gene level expression was quantified as log2(TMP + 1), where TPM is transcript per million as calculated by eXpress (version 1.5.1-linux_x86_64)^48^. See the section Data Availability for information on data deposition at the European Genome-phenome Archive.

### Tissue microarrays (TMAs)

Tumor samples were collected, analyzed and processed to generate 2 TMAs as previously described^49^. The TMAs were composed of high-grade serous carcinoma (n = 204), low-grade serous carcinoma (n = 20), serous borderline tumor (n = 15), serous cystadenoma (n = 14), mucinous carcinoma (n = 49), mucinous cystadenoma (n = 12), endometroid carcinoma (n = 43), clear cell carcinoma (n = 29), undifferentiated carcinoma (n = 4), and Brenner tumor (n = 2).

### siRNA knockdown

SMARTpool siRNAs (Dharmacon) against human *EFNA1* (L-006369-00), *EFNA5* (L-011649-00), and non-targeting siRNA (D-001206-14) were transfected in cells using Lipofectamine 2000 (Life Technologies).

### Immunoblotting

Cells were lysed in RIPA buffer (50 mM Tris–HCl pH 7.4, 150 mM NaCl, 1% Igepal CA-630, 0.5% sodium deoxycholate) containing cOmplete protease inhibitor cocktail (Roche), 1 mM sodium orthovanadate (Sigma-Aldrich) and 5 mM EDTA. Conditioned medium was collected, centrifuged at 21,000 *g* for 15 min at 4 °C and processed as the cell lysates. Protein content was assessed with BCA-kit (Pierce). Lysates mixed with 5 × sample buffer [25% v/v glycerol (Sigma-Aldrich), 0.125 M Tris (Sigma-Aldrich), 0.5 M SDS (Sigma-Aldrich), 0.5 mg/ml bromophenol blue (Sigma-Aldrich), 0.1 M DTT (Life Technologies)] were size-fractionated by gradient (4–20%) SDS-PAGE gel (Bio-RAD) followed by transfer to nitrocellulose membranes (Bio-RAD). The non-specific protein binding sites were blocked for 30 min with 5% non-fat milk or 5% BSA (Biowest) and the membranes were probed with primary antibodies overnight at 4 °C. HRP-conjugated secondary antibodies (Dako) were applied for 1 h at room temperature (RT). Bands were detected by applying enhanced ECL chemiluminescence reagent (GE Healthcare) using x-ray films (Fujfilm) and developed in a Protec OPTIMAX 2010 film processor.

### Immunohistochemistry

The staining of TMAs and bulk tumor sample was performed with automated immunostaining device BenchMark XT (Roche Diagnostics/Ventana Medical Systems) using Ultraview Universal DAB Detection Kit (Roche Diagnostics/Ventana Medical Systems).

### Immunofluorescence

Cells were fixed with 4% paraformaldehyde for 20 min and subjected to blocking and permeabilization with 5% BSA in PBS containing 0.1% Triton X100 for 30 min at RT. Cells were incubated for 1 h in pre-titrated dilutions of primary antibodies in blocking buffer, followed by thorough washes with PBS, and incubation with Alexa-conjugated secondary antibodies for 30 min. Cells were subsequently washed and mounted with Vectashield (Vector Laboratories) containing DAPI. To enhance nuclear PAX8 staining, cells were post-fixed with absolute ethanol for 45 s before blocking.

### Image analysis and statistics

Quantitative assessment of immunoblots was performed using ImageJ. Immunofluorescence images were obtained using Zeiss AxioImager.Z1 microscope outfitted with an ApoTome optical sectioning device and a 20 × Plan Apochromat air objective. Images were processed using Zen 2012. Quantification was performed with CellProfiler 3.0.0., by using DAPI-stained nuclei as primary objects, then propagating the cytoplasmic area from nuclei using a membrane-localizing marker to map the edges of cytoplasm, and finally forming the analysis area by subtracting the nuclear area from each cytoplasm. Mean signal intensities were then obtained from each image and averaged for each treatment/condition. The experimental protein and RNA analyses were performed at least in triplicates, as indicated in the corresponding figure legends, and the statistical significance was determined using two-sided Student t-test. *p* values are depicted as *p < 0.05; **p < 0.01; ***p < 0.001.

For the TMA scoring, a 4-point scale (0: negative, 1: weak, 2: intermediate, and 3: strong) was used to evaluate the maximum intensity of ephrinA5 staining in each core. The scoring was performed independently and in a blinded manner by two investigators, and in case of disagreement, a consensus score was established. Correlation analyses from TMA data were performed through Pearson chi-square test.

The 2011 Agilent 244 K microarray-sequenced HGSC TCGA dataset^35^ was used for the survival association analysis. Overall survival (OS) was defined as the interval from the date of initial surgical resection to the date of last follow-up or death, in months. Progression-free survival (PFS) was defined as the timeframe from the date of initial surgical resection to the date of progression, recurrence, or last follow-up, in months. Cases that were healthy (n = 8), from different primary site than ovary/fallopian tube (n = 4), low-grade (n = 87), or with no information regarding histology or neoadjuvant chemotherapy (n = 48) were excluded from the analysis. Differences in 5-year and 13-year OS, as well as PFS in 40% highest versus 40% lowest Eph-receptor/ephrin expressing patients were estimated using log-rank test. The 40% cutoff was adopted to include the maximum number of samples while minimizing the noise produced by ambiguous samples. *K*-means clustering was also used for analyzing 5-year OS. Cox multivariate analysis integrating the variables age at diagnosis (median cutoff of 59 years), residual tumor after surgery (no vs yes) and FIGO stage (I–IIIB vs IIIC–IV) was also performed to evaluate 5-year OS. TCGA statistics were performed in SPSS, versions 24.0 and 26.0.

The *curatedOvarianData*, a manually curated data collection of microarray data for altogether 2970 OC patients from 23 studies with documented clinical metadata, was used to validate our Eph-ephrin survival association results in a broader OC setting^36^. The datasets were filtered to include only those that had information on vital status, time to death/last follow up, residual tumor after surgery and FIGO stage, and contained at least 60 cases and expression for at least 1000 genes. The TCGA cohort (both the RNA sequencing and microarray datasets) in *curatedOvarianData* was excluded to generate the validation cohort. From the selected datasets (n = 7), cases that complied with sample type (tumor), histology type (serous), stage (late) and grade (high) were included in the analyses. The batch corrected mRNA expression of the 8 ephrin ligands and 14 Eph receptors in these filtered datasets (n = 815 patients) was plotted in a heat map using Heatmapper^50^ and compared to the expression from the TCGA cohort (including both RNA sequencing and microarray data). Multivariate analyses considering residual tumor after surgery (optimal vs suboptimal debulking) and FIGO stage (I to IV) were performed using RStudio. The hazard ratios with 95% confidence interval (CI) and the corresponding significance for all Eph-ephrin associations to OS were obtained and plotted in forest plots using RStudio.

The Affymetrix U133 + 2 mRNA sequencing data from the Cancer Cell Line Encyclopedia (CCLE) was obtained from the dataset uploaded on 29th of September 2012 and used for the Eph/ephrin expression analysis in the cell lines. The probe IDs are collected in Table 1.

**Table 1:** CCLE probe IDs.

Description	Probe ID			
EPHA1	205977_s_at	215804_at		
EPHA2	203499_at			
EPHA3	206070_s_at	206071_s_at	211164_at	
EPHA4	206114_at	227449_at	228948_at	229374_at
EPHA5	215664_s_at	216837_at	237939_at	
EPHA6	1561396_at	233184_at		
EPHA7	1554629_at	206852_at	229288_at	238533_at
EPHA8	1554069_at	231796_at		
EPHA10	1553371_at	236073_at	243717_at	
EPHB1	210753_s_at	211898_s_at	230425_at	
EPHB2	209588_at	209589_at	210651_at	211165_at
EPHB3	1438_at	204600_at		
EPHB4	2028974_at	216680_s_at		
EPHB6	204718_at			
EFNA1	202023_at			
EFNA2	1553573_s_at	208256_at	238956_at	
EFNA3	210132_at			
EFNA4	205107_s_at			
EFNA5	207301_at	214036_at	227955_s_at	233814_at
EFNB1	202711_at			
EFNB2	202668_at	202669_s_at		
EFNB3	205031_at	210883_x_at		

## Supplementary Information

Supplementary Figures.

## Data Availability

All data generated or analyzed during this study are included in this published article and its supplementary information file. The bulk RNA sequencing data that support the findings of this study are available at European Genome-phenome Archive: EGAD00001006456 (RNA sequencing data of HGSC samples, under the study: EGAS00001004714). Publicly available data were obtained through The Cancer Genome Atlas (http://cancergenome.nih.gov/), Bioconductor (http://www.bioconductor.org/packages/2.12/data/experiment/html/curatedOvarianData.html) and The Cancer Cell Line Encyclopedia (https://portals.broadinstitute.org/ccle). Further details are available from the corresponding author on reasonable request.
