# Supplementary material for: Aggressive and recurrent ovarian cancers upregulate ephrinA5, a non-canonical effector of EphA2 signaling duality
Source: Sci Rep. 2021 Apr 23;11:8856. doi: 10.1038/s41598-021-88382-6 (PMC8065122; doi:10.1038/s41598-021-88382-6)
Supplement: Supplementary file 1 — Supplementary Figures. [file 41598_2021_88382_MOESM1_ESM.docx]

**Aggressive and recurrent ovarian cancers upregulate ephrinA5, a non-canonical effector of EphA2 signaling duality**

Joonas Jukonen, Lidia Moyano-Galceran^†^, Katrin Höpfner^†^, Elina A Pietilä, Laura Lehtinen, Kaisa Huhtinen, Erika Gucciardo, Johanna Hynninen, Sakari Hietanen, Seija Grénman, Päivi M Ojala, Olli Carpén, Kaisa Lehti*

†Equal contribution

*Corresponding author

**
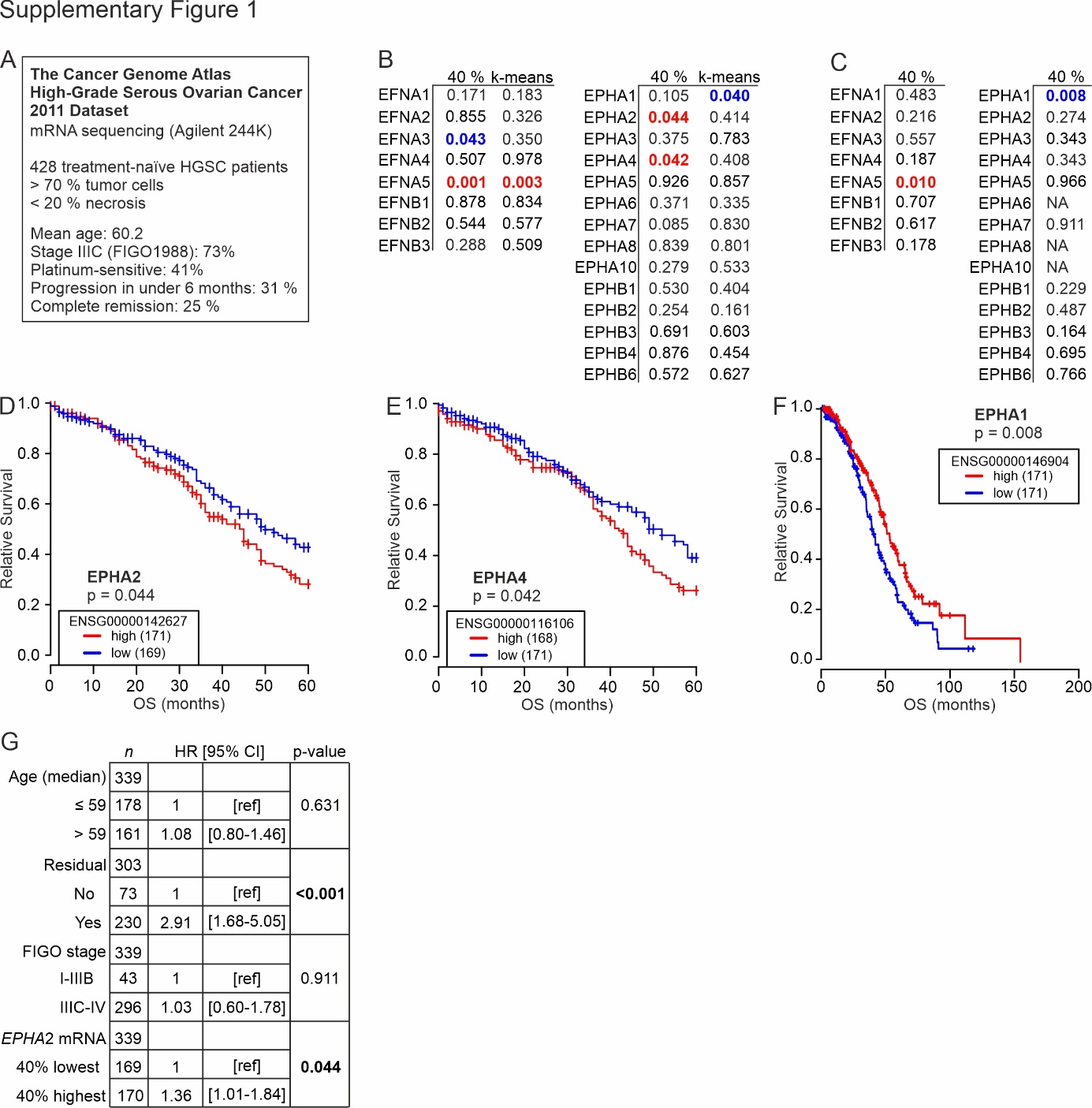
**

**Supplementary Figure 1. Eph receptor and ephrin survival associations in HGSC TCGA dataset.**

(A) Characteristics of the HGSC TCGA dataset.

(B-C) p-values from the logrank tests performed to analyze ephrin and Eph receptor survival associations in HGSC using the TCGA dataset (5-year OS in B and 13-year OS in C). Significant associations between poor OS and low receptor/ligand expression are indicated in blue, and associations with high expression are indicated in red.

(D-F) Kaplan-Meier survival curves illustrate the 5-year or 13-year OS of patients with high or low (top 40% vs. bottom 40%) *EPHA2* (D, 5-y OS), *EPHA4* (E, 5-y OS), and *EPHA1* (F, 13-y OS). Logrank test was used.

(G) Multivariate analysis results for *EPHA2* OS association when considering the variables age at diagnosis (median cutoff of 59 years), residual tumor after surgery (no versus yes) and FIGO stage (I-IIIB versus IIIC-IV). Cox regression was used. HR [95% CI] = hazard ratio with 95% confidence interval.


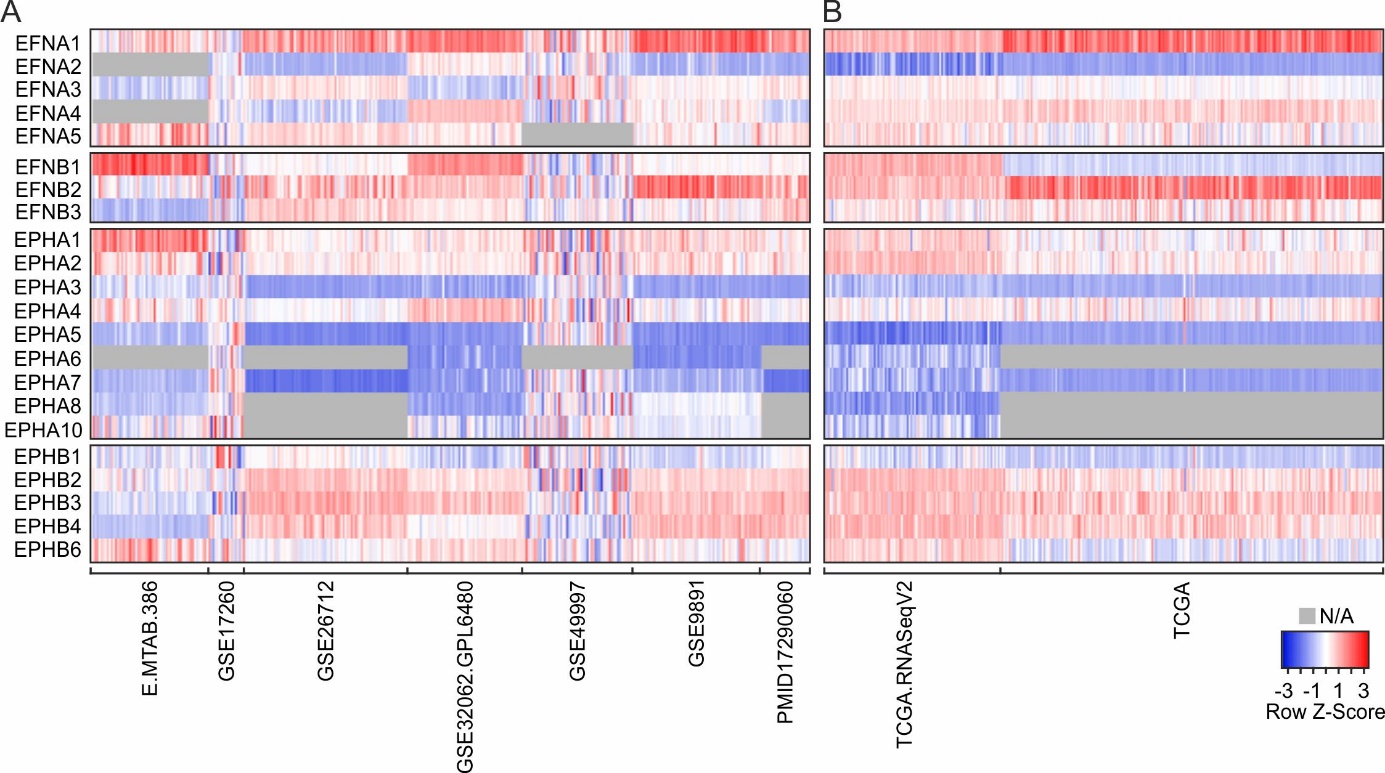


**Supplementary Figure 2. EFN and EPH expression across multiple OC datasets.**

(A-B) Heat map for ephrin ligand and Eph receptor mRNAs in seven independent datasets (A) as well as in TCGA cohort (B; the patient samples overlap in a way that all cases in the RNA sequencing data on the left were included in the microarray data on the right) from *curatedOvarianData*.

**
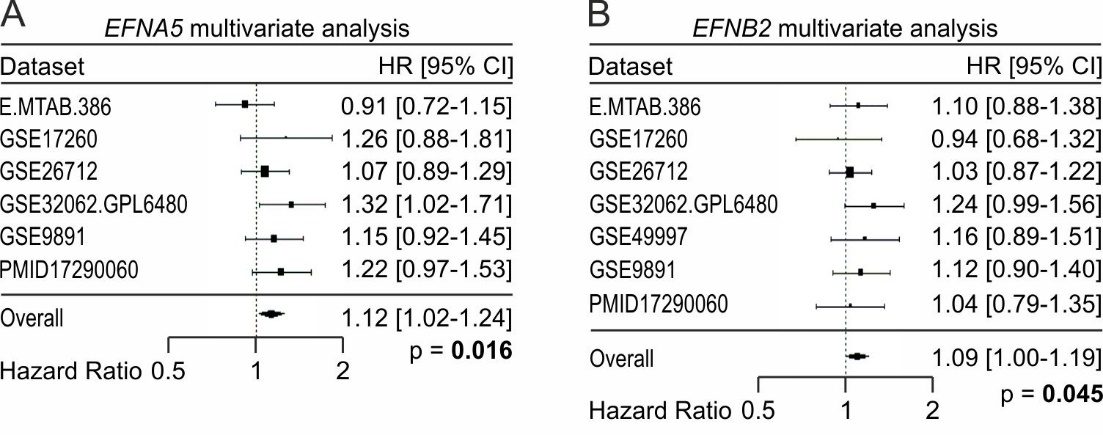
**

**Supplementary Figure 3. Validation of *EFNA5* as well as *EFNB2* ligand survival associations in several independent mRNA datasets of *curatedOvarianData*.**

(A-B) Forest plots for the multivariate analyses of *EFNA5* (A) and *EFNB2* (B) OS associations when considering the variables residual tumor after surgery (optimal versus suboptimal debulking) and FIGO stage (I to IV) in independent OC datasets as well as pooled (overall). Cox regression was used. HR [95% CI] = hazard ratio with 95% confidence interval. N = 6 datasets (A; of note, E.MTAB.386 without individual association to poor survival shows highest overall *EFNA5* expression among the independent datasets, see Supplementary Figure 2A), 7 datasets (B; GSE49997 does not include data for *EFNA5*).

**
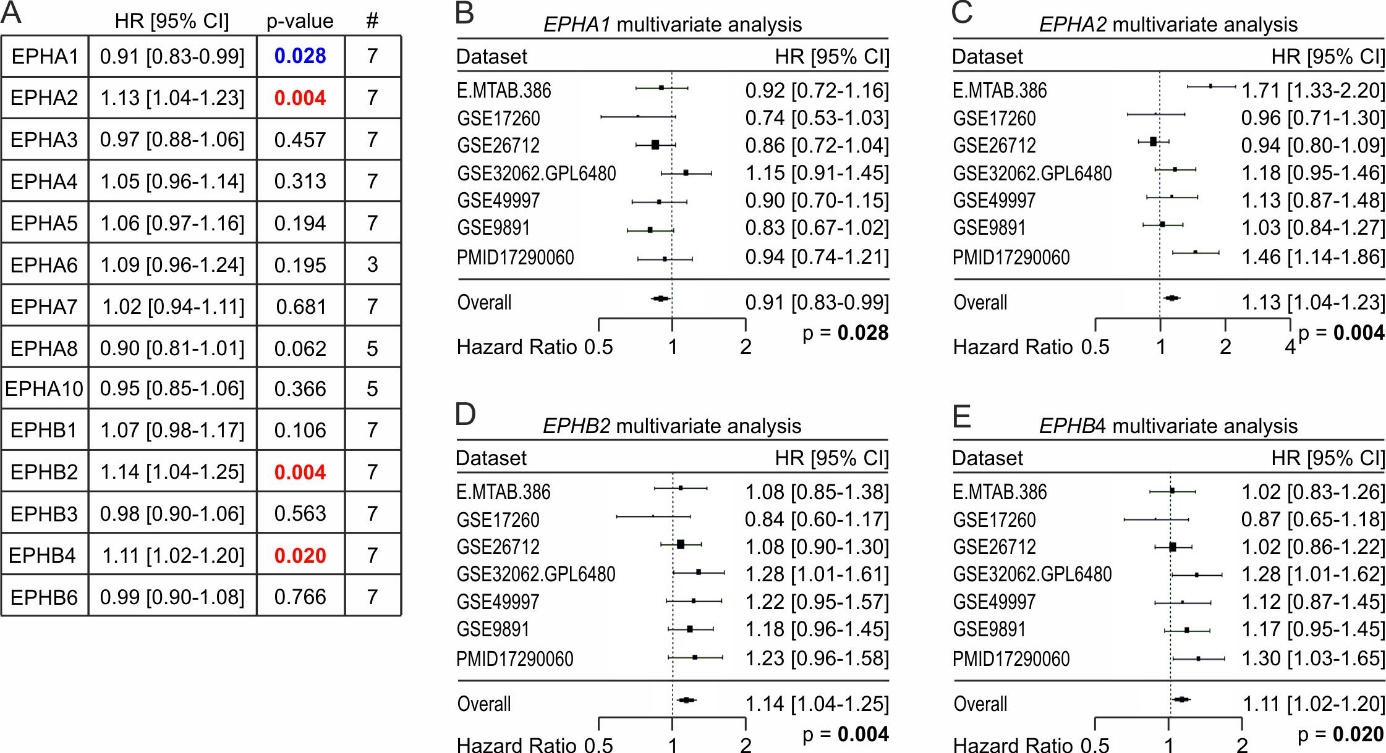
Supplementary Figure 4. Eph receptor survival associations in the *curatedOvarianData*.**

(A) Overall hazard ratios and p-values from the Cox regression tests performed to analyze Eph receptor survival associations in HGSC using the *curatedOvarianData* database. Significant associations between poor OS and low receptor expression are indicated in blue, and associations with high expression in red. HR [95% CI] = hazard ratio with 95% confidence interval, # = number of included datasets.

(B-E) Forest plots for the multivariate analyses of *EPHA1* (B), *EPHA2* (C), *EPHB2* (D) and *EPHB4* (E) OS associations when considering the variables residual tumor after surgery (optimal versus suboptimal debulking) and FIGO stage (I to IV) in independent OC datasets as well as pooled (overall). Cox regression was used. N = 7 datasets.

**
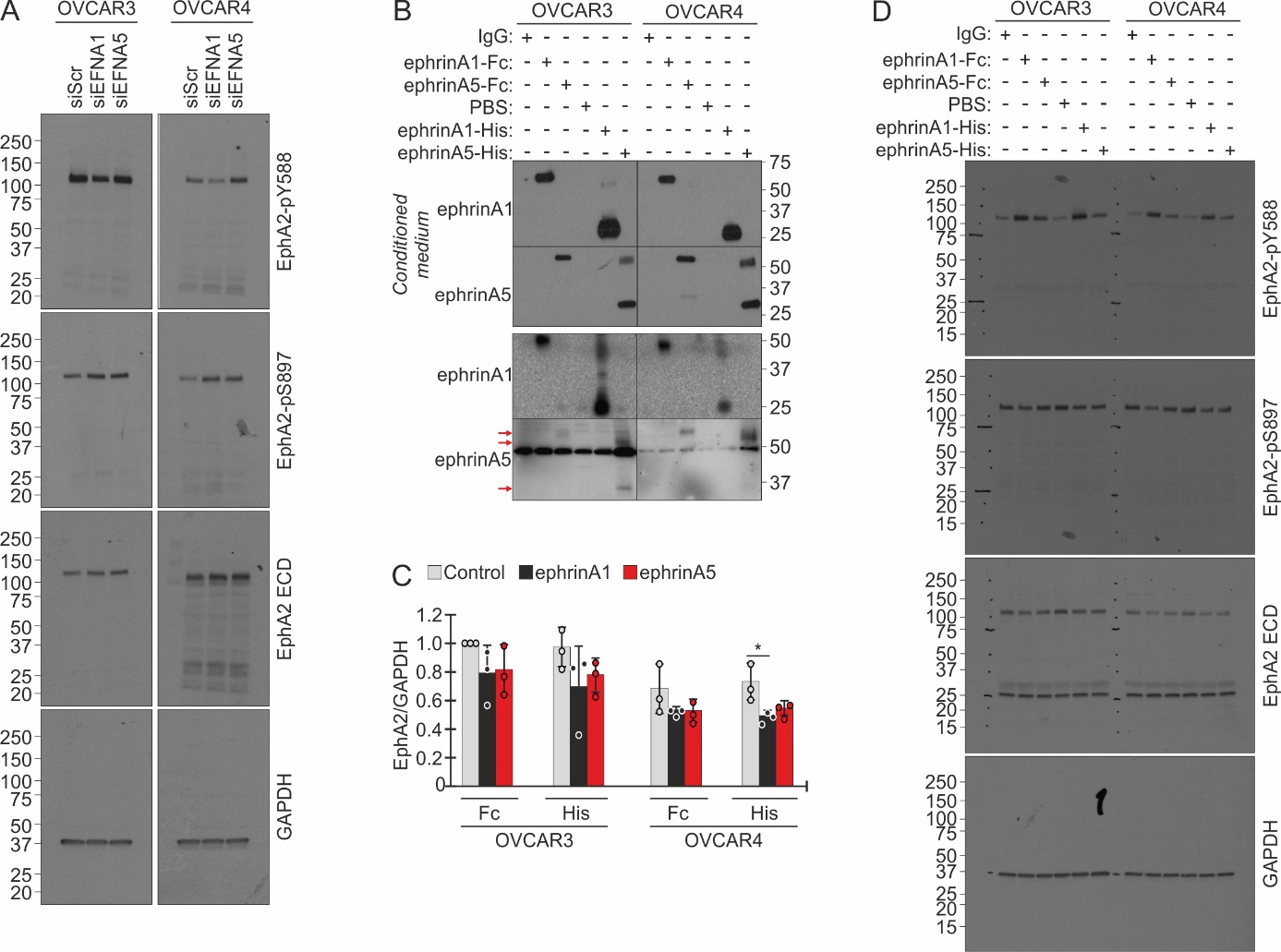
Supplementary Figure 5. Silencing of *EFNA1* and *EFNA5*, and ephrinA1 and ephrinA5 treatments of OVCAR3 and OVCAR4.**

(A) Full-length blots for Figure 2D.

(B) EphrinA1 and ephrinA5 (in conditioned medium or cell-bound) from the same experiment as in Figure 3A were assessed by immunoblotting.

(C) Quantification of EphA2 from the same experiments as in Figure 3B-C. N = 3.

(D) Full-length blots for Figure 3A.

p-values (Student’s t-test): * < 0.05.
